# Supplementary material for: Polyketide synthase gene clusters in the ericoid mycorrhizal fungus Oidiodendron maius: genome-wide prediction, expression profiling, and functional characterization of knockout mutants
Source: Front Plant Sci. 2026 Sep 16;17:1924430. doi: 10.3389/fpls.2026.1924430 (PMC13623903; doi:10.3389/fpls.2026.1924430)
Supplement: Supplementary file 1 [file SupplementaryFile1.zip › Supplementary Materials/Supplementary Folder 1.zip!/Oidma1_AssemblyScaffolds_Repeatmasked/knownclusterblast/cluster3/jgi.p_Oidma1_139674_mibig_hits.html]

| MIBiG Protein | Description | MIBiG Cluster | MiBiG Product | % ID | % Coverage | BLAST Score | E-value |
| --- | --- | --- | --- | --- | --- | --- | --- |
| AAD34554.1 | enoyl\_reductase | BGC0000089 | Lovastatin\_biosynthetic\_gene\_cluster | 43 | 95.9128065395 | 267 | 1.8e-71 |
| ABA02243.1 | dehydrogenase | BGC0000098 | Monacolin\_K\_biosynthetic\_gene\_cluster | 42 | 96.4577656676 | 261 | 1.7e-69 |
| AGO86659.1 | equisetin\_enoylreductase | BGC0001255 | Equisetin\_biosynthetic\_gene\_cluster | 40 | 95.9128065395 | 259 | 4.9e-69 |
| CCT72378.1 | related\_to\_C.carbonum\_toxD\_protein | BGC0001305 | Fujikurins\_biosynthetic\_gene\_cluster | 40 | 94.8228882834 | 250 | 3.9e-66 |
| BAC20562.1 | enoyl\_reductase | BGC0000039 | Compactin\_biosynthetic\_gene\_cluster | 39 | 96.7302452316 | 245 | 7.3e-65 |
| AKC54419.1 | trans\_enoyl-reductase | BGC0001218 | Fumosorinone\_biosynthetic\_gene\_cluster | 37 | 102.72479564 | 227 | 2.7e-59 |
| ADN43684.1 | DmbC | BGC0001136 | Desmethylbassianin\_biosynthetic\_gene\_cluster | 36 | 104.359673025 | 219 | 7.3e-57 |
| CAL69596.1 | hypothetical\_protein | BGC0001049 | Tenellin\_biosynthetic\_gene\_cluster | 35 | 104.08719346 | 213 | 4e-55 |
| BAQ25464.1 | zinc-binding\_dehydrogenase\_family\_protein | BGC0001280 | Betaenone\_C\_/\_betaenone\_A\_biosynthetic\_gene\_cluster | 35 | 95.0953678474 | 176 | 4.2e-44 |
| CBF80481.1 | Enoyl\_reductase\_(Eurofung) | BGC0000959 | Aspyridone\_biosynthetic\_gene\_cluster | 31 | 100.0 | 167 | 2.5e-41 |
